# Diffusion kurtosis imaging, MAP-MRI and NODDI selectively track gray matter myelin density in the primate cerebral cortex

**DOI:** 10.1162/imag_a_00368

**Published:** 2024-11-19

**Authors:** Colin Reveley, Frank Q. Ye, David A. Leopold

**Affiliations:** Wellcome Centre for Integrative Neuroimaging, Centre for fMRI of the Brain (FMRIB), Nuffield Department of Clinical Neurosciences, John Radcliffe Hospital, University of Oxford, Headington, Oxford, United Kingdom; Neurophysiology Imaging Facility, National Institute of Mental Health, National Institute of Neurological Disorders and Stroke, National Eye Institute, National Institutes of Health, Bethesda, MD, United States; Section on Cognitive Neurophysiology and Imaging, Laboratory of Neuropsychology, National Institute of Mental Health, National Institutes of Health, Bethesda, MD, United States

**Keywords:** diffusion MRI, gray matter, kurtosis, myelin, histology, cerebral cortex, marmoset

## Abstract

Diffusion magnetic resonance imaging (dMRI) has been widely used to model the trajectory of myelinated fiber bundles in the white matter. Increasingly, it is also used to evaluate the microstructure of the cerebral cortex gray matter. For example, in diffusion tensor imaging (DTI) of the cortex, fractional anisotropy (FA) correlates strongly with the anisotropy of cellular anatomy, while radial diffusivity (RD) tracks the anisotropy of myelinated fibers. However, no DTI parameter shows specificity to gray matter myelin density. Here, we show that three higher-order diffusion parameters—the mean diffusion kurtosis (MK), the Neurite Density Index (NDI) from neurite orientation dispersion and density imaging (NODDI), and the Non-Gaussian (NG) parameter from mean apparent propagator (MAP)-MRI—each track the laminar and regional myelin density of the primate cerebral cortex in fine detail. We carried out ultra-high-resolution, multi-shelled dMRI in ex-vivo marmoset monkey brains. We compared the spatial mapping of the MK, NDI, and ND diffusion parameters to the cortical myelin distribution of these brains, with the latter obtained in two ways: First, using histological sections finely co-registered to the MRI, and second using magnetization transfer ratio MRI scans (MTR), an established non-diffusion method for imaging myelin density. We found that, in contrast to DTI parameters, each of these higher-order diffusion measures captured the spatial variation of myelin density in the cortex. The demonstration that diffusion parameters exhibit both sensitivity and specificity for gray matter myelin density will allow dMRI to more effectively track human disease, in which myelinated and non-myelinated tissue compartments are affected differentially.

## 1 Introduction

Diffusion magnetic resonance imaging (dMRI) is increasingly used to study the development (Ouyang et al., 2019;Paydar et al., 2014), areal divisions (Avram et al., 2022;Howard et al., 2022;Liu et al., 2018), and pathology (Andica et al., 2020;McKavanagh et al., 2019;Torso et al., 2021) of the human cerebral cortex gray matter in-vivo. However, most investigation into the tissue basis of the dMRI signal has focused on the densely packed axonal projections of the white matter (Johansen-Berg & Behrens, 2013;Song et al., 2002,^2003^,^2005^), where the cell membrane is coated with myelin, a dense composite of lipids and proteins (Kandel et al., 2013;Morell & Quarles, 1999) that presents a strong barrier to the diffusion of water molecules. Much less is known about how dMRI parameters behave in gray matter, which is primarily composed of neuropil, a conglomeration of neurites, glial processes, cell bodies, vasculature, and other unmyelinated structures whose influence on diffusion has yet to be fully established (Jelescu et al., 2020;Novikov et al., 2019). Gray matter also contains a proportion of thin myelinated axons that are enmeshed within the neuropil, and whose structural organization varies by anatomical region and cortical layer (Bock et al., 2011;Braitenberg, 1962;Lewis & Van Essen, 2000;Vogt & Vogt, 1919). These diverse axon arrangements tend to be more diffuse than fiber bundles in the white matter (Lewis & Van Essen, 2000;Vogt & Vogt, 1919), and they may affect the dynamics of diffusing water molecules differently. Moreover, the aggregate dMRI signal in a cortical voxel simultaneously reflects the influence of both neuropil and myelinated axons. Ideally, we would like to distinguish the effects of the myelinated and non-myelinated compartments of gray matter tissue on diffusion, since these two tissue components are affected differently by disease.

Early work showed that the presence of myelin is not necessary for neurites to exhibit diffusion anisotropy (Beaulieu, 2002;Ono et al., 1995) and this was confirmed by dMRI experiments in the developing gray matter, where axons have not yet acquired their myelin sheath (Jespersen et al., 2007,^2012^). Anisotropy occurs partly because the cell membrane reduces diffusivity across the neurites, and partly because there may be greater intrinsic diffusivity in the cytoplasm of neurites compared to the perikaryal cytoplasm or extracellular medium, so that diffusion is inherently faster along axons or dendrites (Beaulieu, 2002;Song et al., 2003). At the same time, foundational studies revealed that the presence of the myelin sheath around axons elevates anisotropy by further reducing diffusivity across their membranes (Jito et al., 2008;Song et al., 2002,^2005^). These early results were obtained in isolated axons, or in selected regions of white matter tissue where large-caliber, myelinated neurites are uniformly aligned. However, myelinated axon fiber bundles traverse much of the human forebrain in all three spatial axes, so that diffusion anisotropies within them induce a heterogenous white matter vector field. This complicates the anatomical interpretation of the diffusion tensor because bundles often intersect within a white matter voxel (Stikov et al., 2011;Wheeler-Kingshott & Cercignani, 2009). More complex models of the dMRI signal often approach this problem by introducing assumptions that are specific to the white matter (Behrens et al., 2007;Zhang et al., 2012).

The mixture of neuropil and myelinated axons in cortical gray matter is a different and highly complex anatomical regime. It is a varied, yet structured environment for which the appropriate modeling assumptions have yet to be fully established (Jelescu et al., 2020;Novikov et al., 2019). For example, myelinated axons do not constitute the bulk of gray matter tissue (Schüz et al., 2002), nor do they form tightly packed bundles that intersect at unpredictable angles; nevertheless, the largest myelinated axons and non-myelinated dendrites often form a variety of interleaved columnar structures that run vertically from the white matter to the pial surface (Peters, 2010;Peters & Sethares, 1996;Rockland & Ichinohe, 2004), along which cell bodies tend to cluster (Buxhoeveden & Casanova, 2002;Peters, 2010). In contrast to the heterogenous vector field in the white matter, diffusion anisotropies in the gray matter tend to produce a vector field whose orientations are uniformly vertical, which may be connected to this columnar anatomy (Jespersen et al., 2010,^2012^;McNab et al., 2013;Reveley et al., 2015).

In a recent study, we examined the relationship between the diffusion tensor and several gray matter histological parameters (Reveley et al., 2022). We found that the fractional anisotropy (FA) showed a very weak correlation to histological myelin density; however, it did track anisotropy in the cellular anatomy of the cortex, suggesting that neuropil influences gray matter diffusion. We found that radial diffusivity (RD) showed some correlation to cortical myelin density as might be expected (Song et al., 2002,^2005^); however, RD was also strongly affected by the arrangement of myelinated axons in the tissue (Reveley et al., 2022). No tensor parameter tracked myelin density specifically, and separately from the arrangement of axons in the gray matter tissue. A number of non-diffusion MRI strategies have been developed to map myelin density in the cerebral cortex. These include the T1w signal (Bock et al., 2011;Glasser et al., 2014), myelin water fraction (Heath et al., 2018;Lazari & Lipp, 2021;Mancini et al., 2020), and the magnetization transfer ratio (MTR) (Grossman et al., 1994;Heath et al., 2018;Koenig, 1991;Lazari & Lipp, 2021;Mancini et al., 2020). Magnetization transfer imaging estimates the density of protons bound to large macromolecules based on the magnetization transfer from the free pool of protons to the bound (Grossman et al., 1994;Underhill et al., 2009;Wolff & Balaban, 1989). Previously, we found that the MTR signal was strongly correlated with myelin density in co-registered histological sections of the gray matter (Reveley et al., 2022). It would be of both theoretical value and practical benefit to find a diffusion parameter that also has this property.

The tensor model assumes that the distribution of diffusing molecule displacements is Gaussian. However, the physical barriers imposed by the tissue might yield different molecule displacement distributions. Since myelin forms a very strong barrier to diffusion, it may be that higher-order diffusion parameters beyond the diffusion tensor are strongly correlated to myelin levels in gray matter. Multiple modeling approaches have been developed to assess non-Gaussian diffusion profiles for dMRI data acquired with multiple b-values. The most conceptually straightforward of these is diffusion kurtosis imaging (Jensen et al., 2005), which offers scalar parameter maps of mean, radial, and axial kurtoses, summarizing deviation from a Gaussian displacement distribution. Previous studies of the white matter have reported a link between these parameter maps and myelin density (Falangola et al., 2014;Guglielmetti et al., 2016;Kelm et al., 2016). Kurtosis measures have also been linked to tissue “complexity” (Wu & Cheung, 2010) as well as to changing tissue constituents during cortical development (Jespersen et al., 2007;Ouyang et al., 2019) and a range of pathological conditions, including astrogliosis, glioma, stroke, and neurodegeneration (Zhuo & Gullapalli, 2020) that are not directly related to myelin levels. It is not yet fully established what tissue properties diffusion kurtosis, and other higher-order diffusion parameters, track in healthy adult gray matter.

Here, we investigate the relationship of diffusion parameters derived from a multishelled dMRI acquisition to the myelin distribution within the cortical gray matter, focusing on the mean diffusion kurtosis (MK) but also including the NG parameter map from the MAP-MRI (Özarslan et al., 2013) model and the NDI map from NODDI (Zhang et al., 2012). We acquired multi-shelled dMRI scans of the fixed brains of two common marmosets (Callithrix jacchus) at isotropic spatial resolutions of 80–150 µm. Tissue myelin density was estimated with MTR scans in the same brains at 75–80 µm. We subsequently sectioned, stained for myelin, and non-linearly co-registered the tissue of one of the brains using a careful, manually guided process, which allowed for a pointwise comparison of the dMRI parameter maps and tissue myelin levels across the gray matter. We found that MK, as well as the NODDI NDI map and the MAP-MRI NG parameter, closely tracked the myelin content in the cerebral cortex, as assessed both by direct histological comparison and comparison to precisely registered MTR data, used as a surrogate for myelin.

## 2 Methods

### 2.1 Methods summary

The postmortem brains of two adult marmosets “case M” and “case P”, both used in previous studies (Liu et al., 2018,^2020^;Reveley et al., 2022), were employed in this work. Both animals were scanned for multi-shelled diffusion MRI (dMRI) and for Magnetization Transfer Ratio (MTR) MRI on the same 7T Bruker preclinical scanner with a 30 cm bore and 450 mT/m gradients. Case M was scanned and then carefully co-registered to an atlas (Paxinos et al., 2013) of the marmoset cerebral cortex as part of the marmoset brain mapping project (Liu et al., 2018,^2020^). The dMRI was obtained at a resolution of 80 µm isotropic in a whole brain scan lasting 15 days, conducted for that project (Liu et al., 2020). The left hemisphere of “case P” was scanned specifically for the present study at a resolution of 150 µm isotropic. As part of a previous study (Reveley et al., 2022), case P was sectioned for Gallyas myelin histology (Gallyas, 1979), which was then non-linearly registered to the dMRI data. All the procedures and provision of materials for this study were in full compliance with the Guidelines for the Care and Use of Laboratory Animals by National Institute of Health and approved by the Animal Care and Use Committee of the National Institute of Mental Health.

### 2.2 Ex-vivo high-resolution MRI acquisition

#### 2.2.1 Case M

Detailed scan information can be found inLiu et al. (2020). Briefly, the brain of case M (male common marmoset, 4.5 years old “Case M”) was soaked with 0.2% gadolinium (1 mmol ml^–1^Gadavist, Bayer) in 1× PBS for 2 weeks and then with 0.2% gadolinium in pure water for 1 day. Case M was then scanned using a coil specially tailored for ex-vivo scanning of marmoset brains. 204 dMRI volumes were acquired at 80 µm isotropic resolution in four shells (8 b = 0, 6 b = 30, 64 b = 2,400, and 126 b = 4,800). The pulse width for the diffusion weighting gradient was 6.4 ms and pulse separation was 14 ms, for a diffusion time of approximately 14 ms. The scan time was 15 days.

MTR data were acquired was acquired for case M, also at 80 µm isotropic resolution using a 3D FLASH sequence. Five MTR scans were acquired, each comprising two volumes with (M_sat_) and without (M_off_) an offset magnetization transfer (±2,000 Hz off-resonance, Gaussian-shaped), which were averaged before calculating the MTR. The MTR value is calculated as 100(1-M_sat_/M_off_). All five MTR scans were then averaged. The total acquisition time was 50 hours.

#### 2.2.2 Case P

Before MRI scanning, the formalin-fixed marmoset brain of case P was soaked with 0.15% gadopentetate dimeglumine (Magnevist, Bayer Leverkusen, Germany) for 3 weeks to reduce the T1 relaxation time as described in a previous study (Liu et al., 2018;Reveley et al., 2022). In order to improve SNR, brain P was cut through the midline. Only the left hemisphere was used in the present study. The left hemisphere was scanned with a 25-mm birdcage volume coil (Bruker). The imaging parameters were: TR = 470 ms, TE = 34 ms, flip angle = 90°, FOV = 38.4 x 24 x 21 mm, matrix size = 256 x 160 x 140, and resolution = 0.15 mm isotropic. The pulse width for the diffusion weighting gradient was 8 ms and pulse separation was 18 ms, for a diffusion time of approximately 18 ms. In order to keep the echo time short, 10 segments were used in the EPI acquisition. The DWI data were acquired using 2 averages, and the acquisition time for each 3D volume was about 22 minutes. In total, 142 DWI volumes were collected to sample the q-space on 5-shells. The 5 shells were defined by b-values: 419, 1,677, 3,773, 6,708, and 10,481 s/mm², obtained by setting the gradient magnitude at 80, 160, 240, 320, and 400 mT/m. 3, 21, 21, 37, and 58 volumes were collected in each shell respectively. Two volumes were collected at b = 0.

The left hemisphere of case P underwent scanning for MTR at 75 µm as described in a previous study (Reveley et al., 2022). Briefly, the MTR images were collected with a 3D FLASH sequence, FOV = 34.8 x 20.4 x 20.1 mm, and matrix size = 464 x 272 x 268. As with case M, the magnetization transfer was achieved in M_sat_scans by a Gaussian-shaped pulse at +2,000 Hz offset frequency in one scan and at -2,000 Hz in the other scan of a paired acquisition. This magnetization transfer pulse was turned off in M_off_scans. The MTR value was calculated as 100(1-M_sat_/M_off_). The number of averages was 5. The total MTR acquisition time for the left hemisphere of case P was about 60 hours.

### 2.3 MRI processing

Here, we describe MRI pre-processing steps and computation of diffusion tensor, kurtosis, NODDI, and MAP-MRI parameters in each ex-vivo brain.

#### 2.3.1 Case M

We used the publicly available 80 µm MTR and dMRI data from case M, which was previously preprocessed (Liu et al., 2020) using the DIFFPREP of TORTOISE (Pierpaoli et al., 2010). We removed the left hemisphere and closely cropped the right hemisphere using fslroi, part of FSL 6.0.5.2 (Woolrich et al., 2009), to save computation, since only the right hemisphere was aligned to the atlas labels. We calculated the mean, axial, and radial kurtoses of the 80 µm data using dtifit part of FSL 6.0.5.2 (Woolrich et al., 2009). For the remaining parameter maps, we downsampled the diffusion data from 80 µm to 160 µm using the “fslmaths -subsamp2offc” command in FSL 6.0.5.2 (Woolrich et al., 2009). We computed the mean kurtosis a second time with “RobustDKIFitting” MATLAB script (Henriques et al., 2021) and compared this to the DTIFIT results, finding good agreement. We computed the NODDI model using AMICO (Daducci et al., 2015) version 1.5.4 with default diffusivity parameters and the “ex-vivo” parameter set to 1. We estimated the MAP model order 4 using TORTOISE (Pierpaoli et al., 2010) version 3.2.0, EstimateMAPMRI with default parameters.

#### 2.3.2 Case P

For case P, we pre-processed the data using the DIFFPREP of TORTOISE (Pierpaoli et al., 2010). We registered MTR data to the histology slice plane and as described in a previous publication (Reveley et al., 2022), and performed a rigid body (6 parameter) registration of the dMRI data to the MTR using FSL 6.0.5.2 FLIRT (Woolrich et al., 2009), thus upsampling the dMRI from 150 to 75 µm with spline interpolation. We estimated the MAP model at order 4 using TORTOISE (Pierpaoli et al., 2010) version 3.2.0, EstimateMAPMRI with default parameters. We computed the NODDI model using the NODDI toolbox 1.4 (Zhang et al., 2012), with parameters disoIdx 2.0E-9, diIdx 0.4E-9. We calculated the mean, axial, and radial kurtoses of the upsampled 75 µm data using DTIFIT part of FSL 6.0.5.2 (Woolrich et al., 2009), and compared these results to those of the “RobustDKIFitting” MATLAB script (Henriques et al., 2021), finding good agreement.

### 2.4 Anatomical mapping

#### 2.4.1 Case M

The cortex of case M was carefully labeled and aligned to the Paxinos atlas (Paxinos et al., 2013) in previous work (Liu et al., 2018,^2020^). We removed the left hemisphere of the atlas MTR template and atlas labels since these were a mirror image of the right. We fine-tuned the alignment of the atlas labels and MRI data using a shift/translate only FLIRT transform with no interpolation. We used the medium granularity labels of the marmoset brain mapping labels (Liu et al., 2018) in this work. We manually trimmed the anatomical labels at the white/gray and pial boundaries to avoid partial volume effects and non-brain voxels. The white matter voxels were defined as part of the marmoset brain project white matter atlas (Liu et al., 2020). We downsampled each of the three label volumes released as part of the project to 160 µm using the “fslmaths -subsamp2offc” command in FSL 6.0.5.2 (Woolrich et al., 2009). We manually eroded each label slightly to avoid partial volume effects and combined the three volumes into a single mask file.

#### 2.4.2 Case P

For estimation of cytoarchitectural boundaries, we non-linearly registered the right hemisphere of case M MTR to the left hemisphere of case P MTR using ANTS 2.4.1 (Avants et al., 2011), as described in a previous study (Reveley et al., 2022). We then manually entered the coordinates of the region boundaries to the analysis code described below.

### 2.5 Histology acquisition

Myelin histology was obtained and processed from case P only as described in a previous study (Reveley et al., 2022). Briefly, the left hemisphere of case P was sectioned at 50 µm on a freezing microtome. We used the Gallyas silver stain (Gallyas, 1979) which stains individual axons at high resolution. Each section was digitally scanned at 0.88 μm resolution (×10 magnification) using a Zeiss Axioscan microscope slide scanner. Seven Gallyas stained sections were selected for nonlinear registration based on criteria of even staining and minimal damage from a previous study (Reveley et al., 2022).

### 2.6 Histology processing

To estimate the anisotropy content of the myelin histology we employed the structure tensor (ST) method (Budde & Frank, 2012), as described in a previous study (Reveley et al., 2022). Briefly, the 0.88 μm per pixel resolution histology images were processed using the structure tensor as implemented in the “OrientationJ” ImageJ package using a local Gaussian window σ = 85 pixels and image gradients computed with a cubic spline. Since the dMRI data contain diffusion information gathered over a 150 μm cubic area, the histology structure tensor at each pixel was a distance-weighted function of the structure tensors within a radius of 75 μm. For each pixel, the structure tensor coherence was computed from the ST eigenvalues.

### 2.7 Histology registration

The 75 µm MTR was manually rotated to the histology slice plane and the dMRI data registered to this using FLIRT as described above. Histology parameter maps of stain density and anisotropy at 75 µm resolution were then non-linearly registered to the MRI. We performed careful nonlinear alignment of seven local frontal and parietal ROI from the histology to the MRI. Stain intensity maps of the 75 µm maps were first binarized using a threshold adjustment in imageJ (Schindelin et al., 2012) and then registered to their corresponding MRI slices to correct for shrinkage and deformation, first using manual 2D alignment in imageJ and then MIND (Heinrich et al., 2012) MATLAB code (α = 0.1) as described in a previous study (Reveley et al., 2022).

### 2.8 Data analysis

#### 2.8.1 Case M

The data inFigure 2were plotted together in MATLAB 2023b at a resolution of 80 µm by concatenating a mask of all the gray matter regions obtained above (“Anatomical Mapping”), while those inFigure 4used 160 µm labels. ForFigure 3, each region label was evaluated separately.

#### 2.8.2 Case P

Once rotated into the histology slice plane, each coronal slice of the dMRI parameter maps acquired for this study was converted to quantitative tif format and integrated into a software system written for a previous study on MRI histology comparisons (Reveley et al., 2022). Briefly, this system took advantage of the fact that cortex consists of vertically oriented tissue components to produce a matrix representation of the non-linearly registered dMRI and histology data whose columns were derived from data sampled along the vertical dMRI tractography and whose rows spanned the horizontal direction. Tractography lines were integrated through the 2 d projected principal eigenvectors in each gray matter ROI. A vector of unique pixels whose coordinates intersected one streamlines was acquired, each of which was then resampled to a fixed length using bilinear interpolation, to yield a simple matrix representation of the gray matter. This operation was invertible such that data in matrix form could be displayed in image space.

Our statistical analysis proceeded by parcellating these matrix representations of the nonlinearly registered histology and MRI data. The parcellation strategy served to separate out regions of potentially differing stain intensity, and to assess relationships between histology and MRI variables in different anatomical regions in a statistically independent manner. For each parcel we took the Pearson correlation between sample variables, in, for example,Figures 5Cand6A. To assess the average laminar properties of histology or dMRI variables, we averaged the rows of the matrix representation and plotted the resulting vector, for example, inFigures 5Band6B.

## 3 Results

Our principal goal was to determine which diffusion MRI (dMRI) parameters most closely track the density of myelin in the gray matter tissue of the cerebral cortex. To this end, we analyzed high-resolution, ex-vivo dMRI data from the brains of two marmoset monkeys which were collectively scanned for over 3 weeks at 7T (seeSection 2). We then compared this dMRI data to the myelin content of the tissue, first as assessed by a non-diffusion MRI sequence, and second as assessed by myelin-stained histology gathered from one brain, and carefully co-registered to the MRI (Reveley et al., 2022). We employed the magnetization transfer ratio as our non-diffusion MRI measure, since this sequence is known to be sensitive to large macromolecules such as those comprising myelin (Grossman et al., 1994;Heath et al., 2018;Henkelman et al., 2001). In previous work, we showed that MTR intensity closely matched the fine-scale distribution of myelin in the cortical sheet, using histology data gathered from the scanned tissue (Reveley et al., 2022).

### 3.1 MTR tracks histological myelin levels in the cortex

**Fig. 1.**
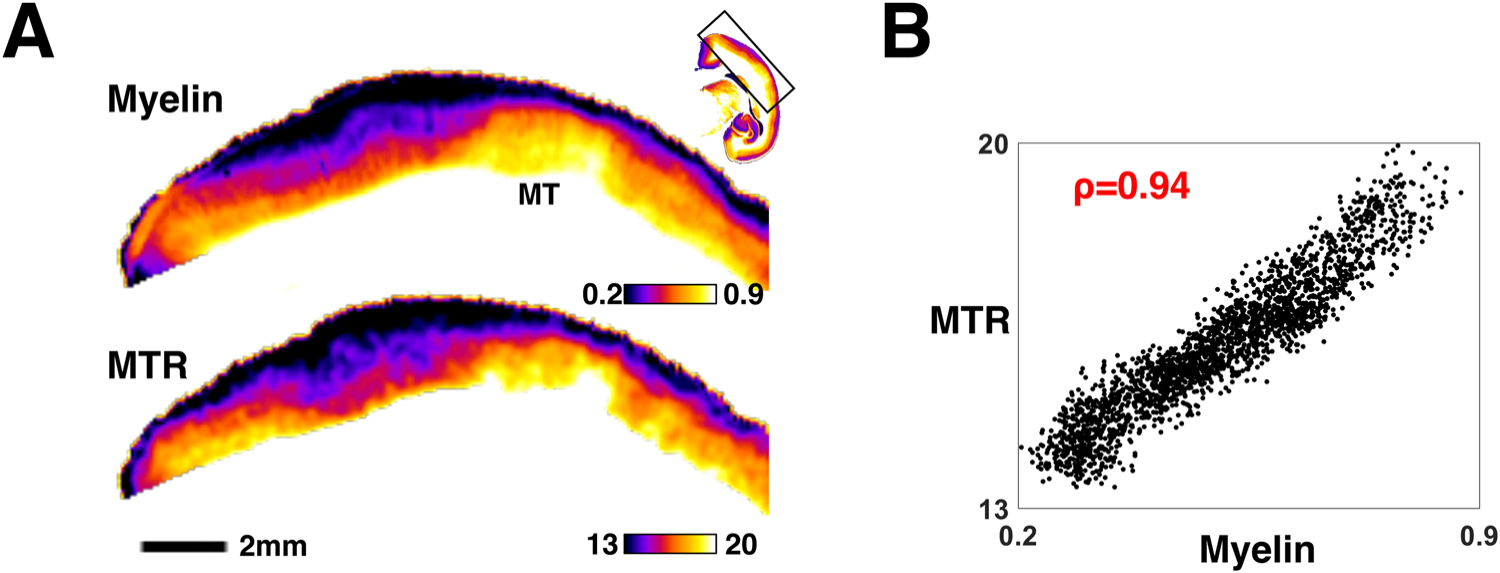
Histological myelin is strongly correlated with Magnetization Transfer Ratio (MTR) MRI. (A) MTR and myelin histology (Gallyas stain) non-linearly registered at 75 µm resolution, showing area MT. Myeloarchitectural features (top row) show strong congruence with MTR intensity (bottom row). (B) Pixelwise scatter plot of the data shown in (A) shows a very strong correlation and linear relationship (ρ = 0.94). Correlations for all seven myelin-stained sections are shown inSupplementary Figure 1.

We began by re-establishing that MTR is a valid surrogate measure for myelin intensity in the cerebral cortex. To do this, we directly compared the spatial distribution of MTR intensity to seven non-linearly registered myelin-stained sections obtained from the scanned brain. This comparison is shown for one segment of the adult marmoset cerebral cortex inFigure 1A, where the myelin-stained histological section (top) has been resampled to match the 75 µm isotropic resolution of the MTR scan (bottom). The myelin in the histological sections and the MTR signal in the MRI data followed a similar gradient, showing highest intensity in deep cortical layers and declining toward the pial surface. In addition to this laminar correspondence, the two modalities also matched in their tangential cortical variation. An obvious example of this is the higher signal intensity in the middle temporal (MT) area, which is known for its high myelin content. For this registered section,Figure 1Bshows a pixel-by-pixel comparison of the two modalities, demonstrating a strong correlation (ρ = 0.94). Applying this analysis to multiple sections, we found a similarly strong correlation between MTR and histological data (Supplementary Fig. 1), confirming that the high-resolution MTR acquisition from the gray matter of fixed brains could serve as a reliable surrogate for cortical myelin distribution.

With this tool in hand, we focused on our target question of which diffusion parameters might accurately reflect cortical myelin content, a potentially important use of dMRI for clinical neurology. Given that neither FA nor RD are effective predictors of gray matter myelin density (Reveley et al., 2022), we investigated the hypothesis that dMRI model parameters reflecting non-Gaussian diffusion may track it more closely. The idea that myelin restricts, rather than merely slows diffusion across axons, has been discussed in previous diffusion studies (Guglielmetti et al., 2016;Jelescu et al., 2016;Jespersen et al., 2010;Kelm et al., 2016). In this study, we compare cortical myelin density captured by MTR and by finely co-registered histology to higher-order diffusion parameters, using an ultra-high spatial resolution that captures the detailed laminar and tangential variation of myelin content across large areas of the primate cortical sheet.

### 3.2 Mean diffusion kurtosis reliably tracks whole-brain myelin levels as measured by MTR

We first compared the MK mapped across the whole cerebral cortex with the myelin content as estimated by the MTR scan. Since both maps are MRI measures from the same ex-vivo sample, the spatial registration was very precise. Importantly, the MK and MTR measures capture fundamentally different physical processes (Henkelman et al., 2001). Nonetheless, we found the two measures exhibited strong similarity in the monkey cortex.

**Fig. 2.**
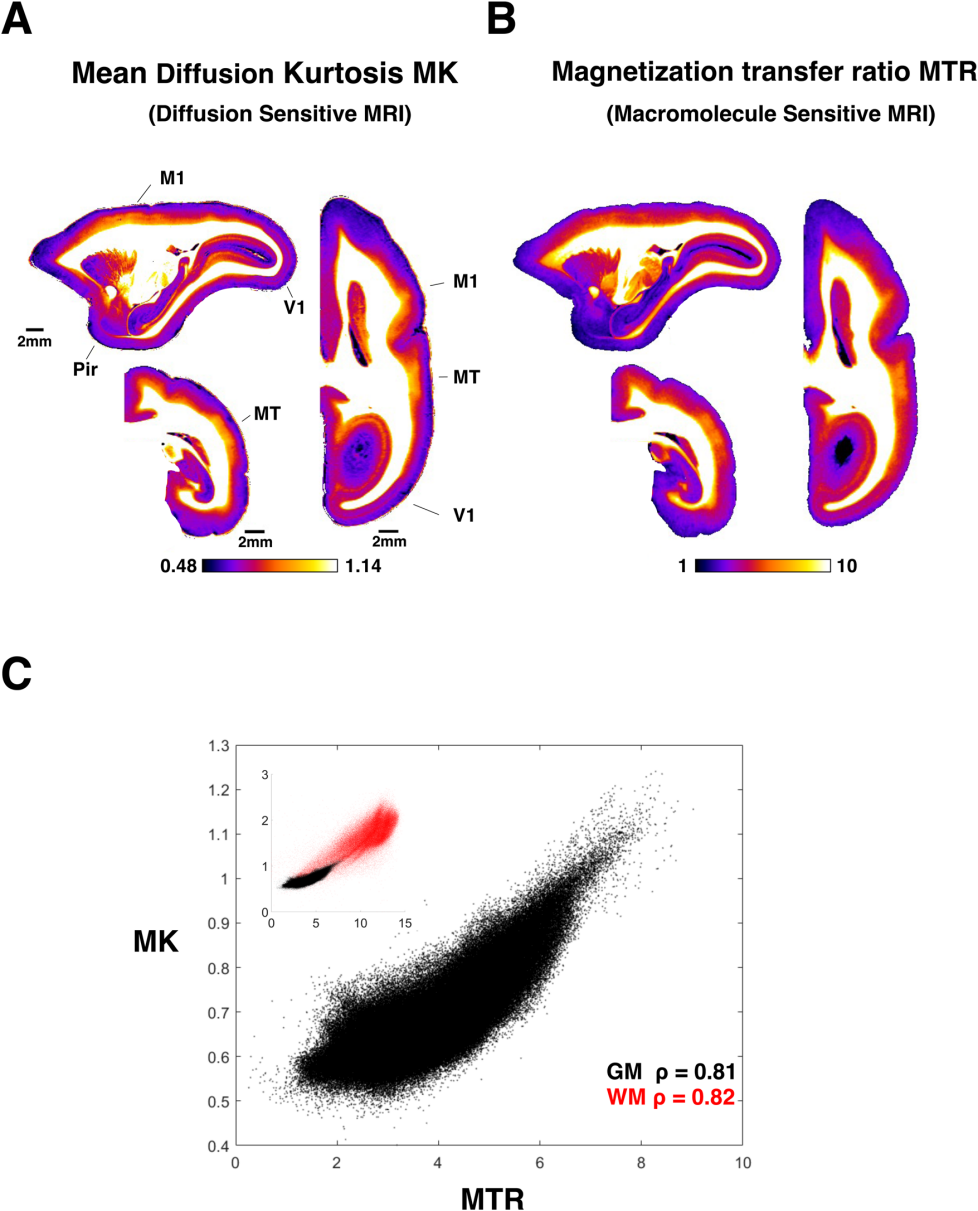
Mean diffusion kurtosis and the magnetization transfer ratio have a similar spatial profile. (A) The distribution of mean kurtosis at 80 µm isotropic resolution matches the expected myelin distribution in highly myelinated (Primary motor**M1**, middle temporal**MT**), moderately myelinated (Primary vision**V1**), and lightly myelinated (Piriform cortex,**Pir**) cortical regions. (B) The distribution of MTR is visually similar to MK, especially in high myelin areas. (C) Intensity of MK and MK voxels plotted against each other in gray matter (black, ρ = 0.81) and white matter (red, ρ = 0.82). Inset shows gray matter and white matter together.

Figure 2A and Billustrate this similarity in representative coronal, sagittal, and axial slices at 80 μm isotropic resolution. Within these sections, the close correspondence in both the laminar and tangential features is evident. In the laminar direction, the MK and MTR measures both exhibited the expected myelin gradient, showing high values in the lower layers near the white matter and decreasing values toward the pia. In the tangential direction, the changes across cortical areas were also similar. For example, the elevated MTR in MT (middle temporal cortex) and M1 (primary motor cortex) were reflected by elevated MK in the same areas, perhaps due to putative restricted diffusion within the relatively more numerous axonal myelin sheaths in these cortical regions.

We quantified the pixelwise relationship between MK and MTR (Fig. 2C), which revealed a strong correlation in the gray matter (ρ = 0.81, blackFig. 2C). The mean kurtosis measure, therefore, appeared sensitive to variation in the vertical and horizontal cortical myelin distribution, in a similar way to the MTR. Interestingly, when this analysis was extended to the white matter, with its higher overall myelin content due to dense composition of myelinated axons, the correlation remained strong (ρ = 0.82 red insetFig. 2C). White matter pixels with higher MTR values, reflecting a higher spin magnetization transfer from the bound pool of protons to the free pool water protons, also exhibited higher MK, reflecting more restricted water diffusion. This robust agreement between diffusion and large-molecule MRI measures in the precisely registered maps of the same brain suggests that mean diffusion kurtosis is a strong marker for myelin content. At the same time, the strength of the gray matter correlation was slightly weaker than that between myelin histology and MTR, shown inFigure 1B. To investigate this discrepancy further, we examined the regional variation in the relationship between MK and MTR.

**Fig. 3.**
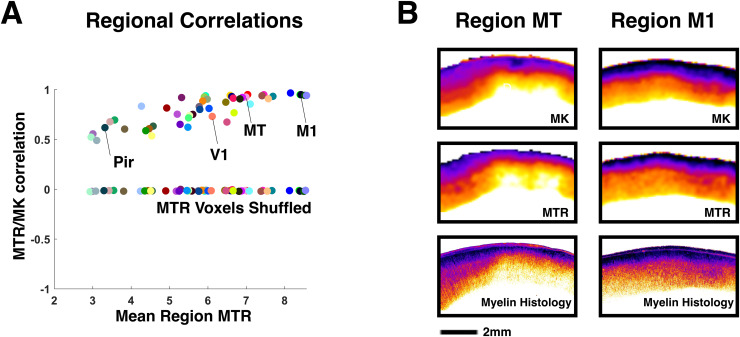
Correlations of MK and MTR at 80 µm resolution for each cortical region. (A) Pearson correlation coefficients between MK and MTR for each marmoset cortical region as delineated inLiu et al. (2018), plotted against the mean MTR value for each region. Higher correlation coefficients were observed for regions with higher mean MTR values. A simple shuffle control was implemented by randomly shuffling the voxel order of the MTR data. See color key inSupplementary Figure 2. (B) MTR, MK, and myelin histology examples from two cortical regions M1 (Primary motor) and MT (middle temporal).

We divided the cortex into anatomical regions based on the areal definitions of the Paxinos atlas (Paxinos et al., 2013) and marmoset brain mapping project (Liu et al., 2018,^2020^), using the registered maps provided by a previous study (Liu et al., 2018,^2020^). We found that the correlation coefficient between MK and MTR varied across cortical regions (Fig. 3A). In contrast to a surface-based analysis of values averaged through the cortical depth, our voxel-based methods at ultra-high-resolution captured rich laminar detail of the cortex (Fig. 3B). Nevertheless, the variation in regional correlation coefficients was related to each region’s total myelin content. For example, in cortical regions where the mean MTR value was high, and which were known to have high myelin content, for example M1 or MT (Fig. 3B) correlation levels approached 1, whereas regions with low MTR, such as the piriform cortex (Pir), exhibited lower correlations, closer to 0.6. The lower correlations may be linked to a decline in contrast relative to noise where myelin content is lower, rather than a failure of either measure to track myelin content (Supplementary Fig. 2).

### 3.3 MAP-MRI and NODDI parameters track mean kurtosis and MTR

We next expanded our investigation of dMRI and cortical myelin to include two further popular diffusion models, namely the non-Gaussian (NG) component of the mean apparent propagator (MAP-MRI) MRI model (Özarslan et al.,^2013^) and the neurite density index (NDI) calculated as part of neurite orientation dispersion and density imaging (NODDI) (Zhang et al., 2012). The NG parameter computed from MAP-MRI is conceptually similar to mean kurtosis. However, NDI is not a measure of non-Gaussian diffusion per se, but is instead part of NODDI, a biophysically motivated tissue model of Gaussian diffusion that seeks to estimate the variation in neurite density (Zhang et al., 2012). In NODDI, contributing gray matter neurites can potentially include both myelinated and unmyelinated axons, as well as dendrites, all of which are modeled as sticks with negligible radial diffusivity (Zhang et al., 2012).

**Fig. 4.**
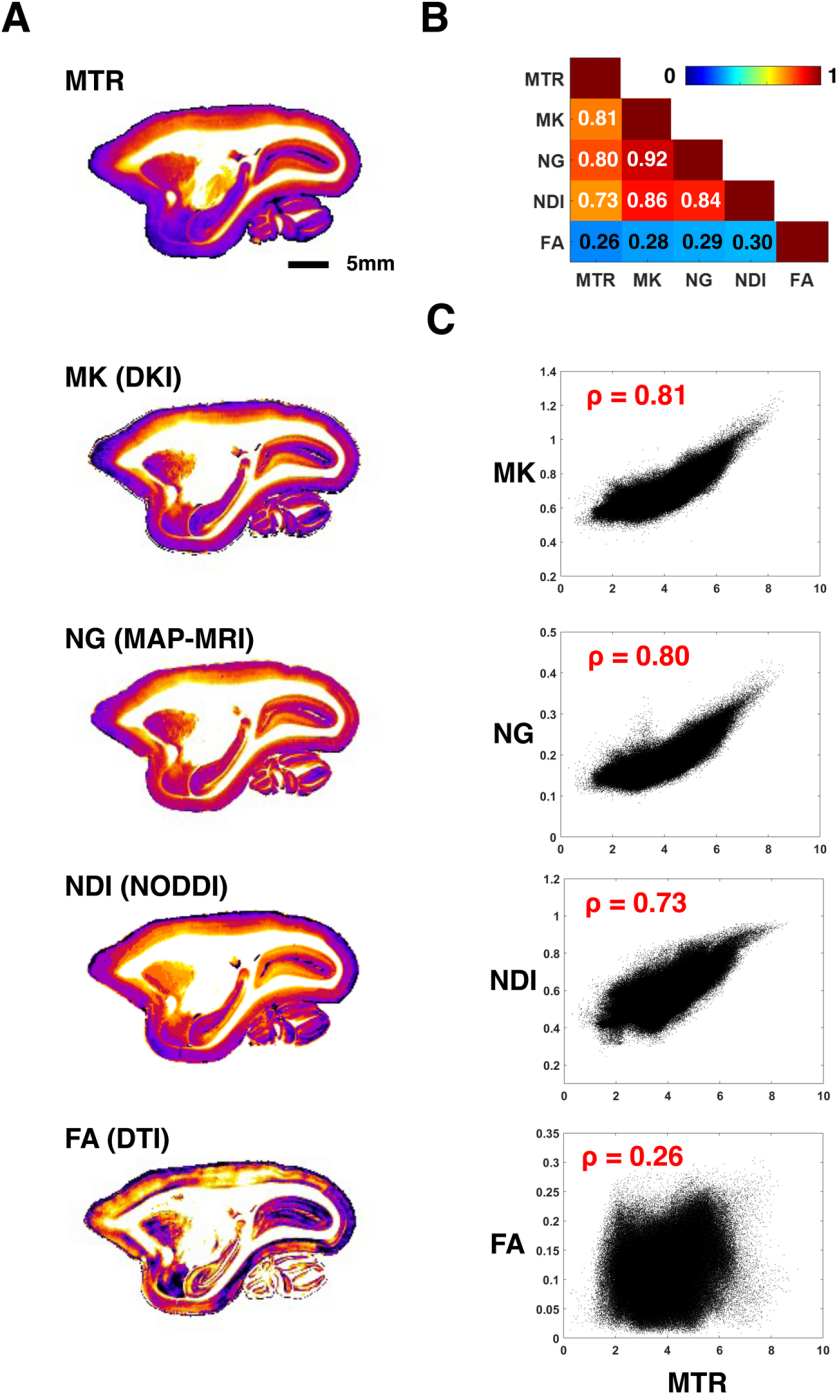
The parameter maps NG from MAP-MRI and NDI from NODDI show a similar relationship to MTR as mean kurtosis (MK). (A) Example slices of MTR, MK, NG, NDI, and FA from the marmoset. The first 4 are very similar in the gray matter, while the FA shows a different cortical profile. (B) Pearson correlation coefficients between all parameter maps are strong, except those involving FA. (C) When plotted against MTR, NG and MK both show a very similar linear relationship. The relationship of NDI to MTR is slightly different, but still qualitatively similar. In contrast, the correlation between FA and MTR is poor and the values appear randomly distributed. The analysis included only gray matter.

We found that all three of the dMRI parameter maps we considered (MK, NG, and NDI) resembled the MTR and one another in the cortical gray matter but differed substantially from the FA maps of the diffusion tensor (Fig. 4A). The correlation between the three dMRI measures of interest was extremely high, with the correlation between MK and NDI at 0.86 and between MK and NG at 0.92. The correlations with each of these parameters to the MTR maps were also high, albeit with the correlation of NDI to MTR taking a slightly lower value (0.73) than those of MK and NG (0.81 and 0.8 respectively). By contrast, the relationship of FA to the three putatively myelin-sensitive dMRI measures, and to MTR, were all much lower, between 0.26 and 0.3 (Fig. 4B). When we examined the spatial relationships of dMRI parameters to MTR, we found that MK and NG exhibited a nearly identical, roughly linear voxel-wise relationship to MTR, and NODDI showed a slightly different but still similar relationship. The relationship of FA to MTR, however, was much weaker (Fig. 4C).

### 3.4 Confirmation using histological myelin content

Having established the close relationship between MTR and the diffusion parameters MK, NG, and NDI over the whole brain, we next focused on a fine-grained comparison of the dMRI parameters with myelin histology obtained from the same brain. We compared seven non-linearly registered sections of myelin histology to matching dMRI and MTR MRI. In the example scatter plots from one section shown inFigure 5A, the MRI parameters of interest all showed a clear linear relationship with histological myelin, with MTR, MK, and NG showing Pearson correlations of 0.85, 0.82, and 0.84 respectively. NDI showed a slightly weaker correlation of 0.75. FA showed a lower overall correlation and a more complex, piecewise relationship to myelin density, which we previously reported (Reveley et al., 2022).

**Fig. 5.**
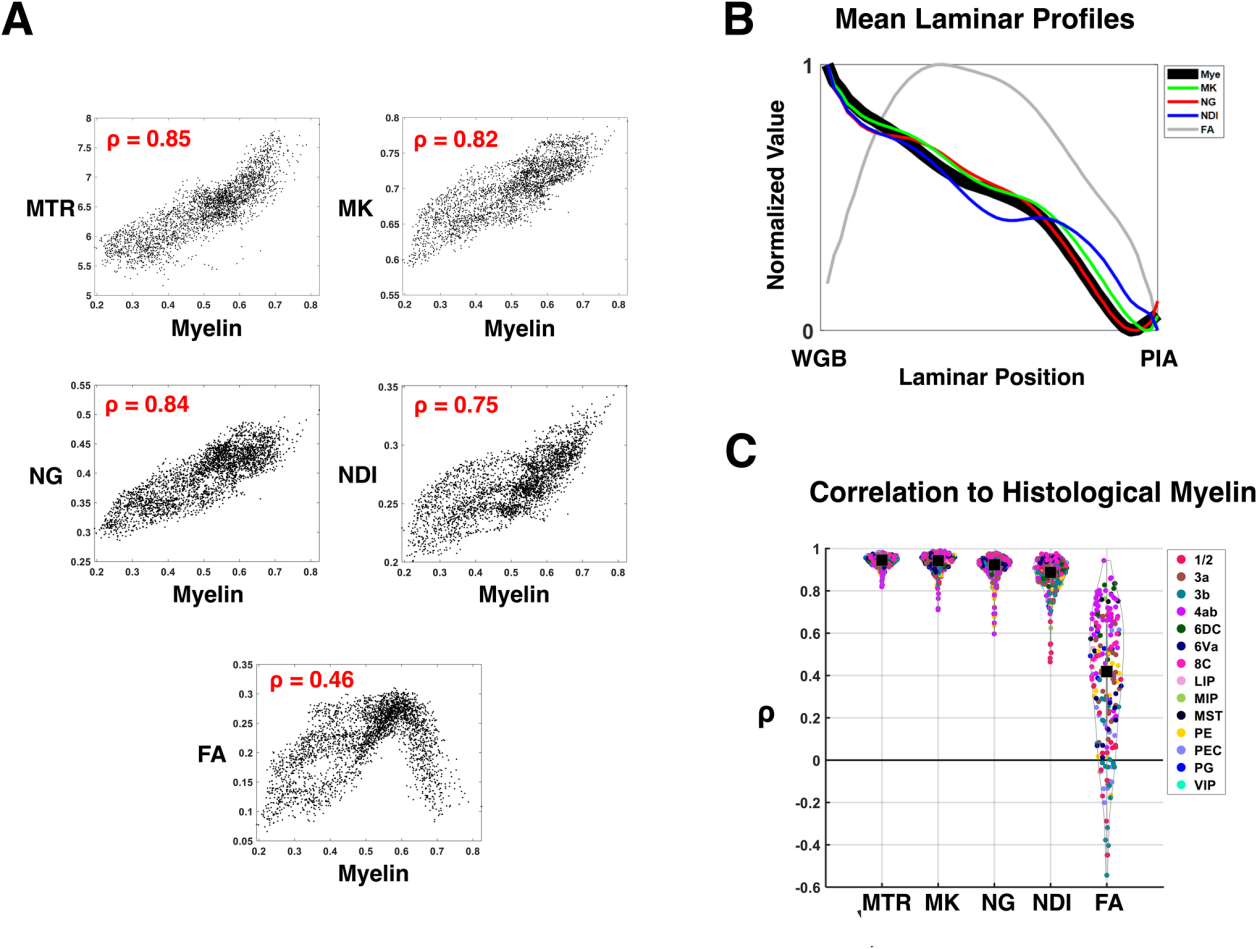
The parameter maps MTR, NG, MK, and NDI show a similar relationship to histological myelin. (A) Pixelwise scatter plots of MTR, NG, MK, NDI, and FA plotted against histological myelin in the gray matter of one histological section. (B) The mean laminar profiles (seeSection 2) of histological myelin, MK, NG, NDI, and FA. All the parameters follow a similar monotonic decline to myelin, except FA. (C) MTR, MK, NG, NDI, and FA cortical parcel correlations to histological myelin (seeSection 2).

Continuing to investigate the histological basis of the dMRI parameters, we next examined their laminar variation, their variation by cortical area, and how both of these tracked histological myelin content. To assess the similarity in laminar variation, we sampled the cortex along columnar lines derived from 2D tractography and resampled the resulting data into a matrix formulation (seeSection 2). This allowed us to calculate and compare the mean laminar profile of the various MRI measures to the co-registered myelin histology (Fig. 5B). We found that NG and MK exhibited a laminar profile that was nearly identical to that of myelin intensity, declining monotonically from deep to superficial layers. However, as expected, the laminar profile of FA differed substantially from that of myelin, and from the other diffusion parameters, with minima at the white/gray and pial boundaries and a peak in middle cortical layers. This laminar profile reflects the influence of neurite organization as well as density, and non-myelinated components as well as myelinated (Reveley et al., 2022).

To provide a more granular analysis, we next sampled the matrix representation of the cortex into columnar parcels (Reveley et al., 2022) running vertically from the white matter to the pia and with a horizontal width of about 300 µm (Fig. 5C). Within each of these parcels, we measured the correlation of the MRI parameters with histological myelin. This analysis showed a high spatial correlation of voxels within most columnar parcels, with the highest median values observed for MTR (0.95) and MK (0.94), slightly lower values for NG (0.92) and NDI (0.89), but much lower values observed for FA (0.42).

Together, these analyses reveal that the diffusion-based measures—obtained either by modeling the spatial distribution of diffusion (MK, NG) or by biophysical forward modeling of the neurite distribution (NDI)—were all good measures of the cortical myelin distribution. This result is especially clear when the higher-order parameters are contrasted to FA, which also reflects the influence of non-myelinated components in gray matter.

### 3.5 Radial diffusivity tracks myelin organization

The above results show that several higher-order models obtained from multi-shelled diffusion data closely track myelin content in the cortex. Although FA in the cortex is not specific to myelinated structures (Beaulieu, 2002;Reveley et al., 2022), radial diffusivity (RD), another measure derived from the diffusion tensor, has shown more specificity to myelin in both gray (Reveley et al., 2022) and white matter (Song et al., 2002). In some studies, RD has been shown to correlate with histological measures of myelin content and has been used as a marker for assessing myelin integrity and alterations in white matter microstructure (Lazari & Lipp, 2021;Mancini et al., 2020). Previously, we found that RD tended to track myelin organization rather than myelin intensity in gray matter (Reveley et al., 2022). We next examined the relationships between RD and myelin, using the same approach as above (Fig. 5).

**Fig. 6.**
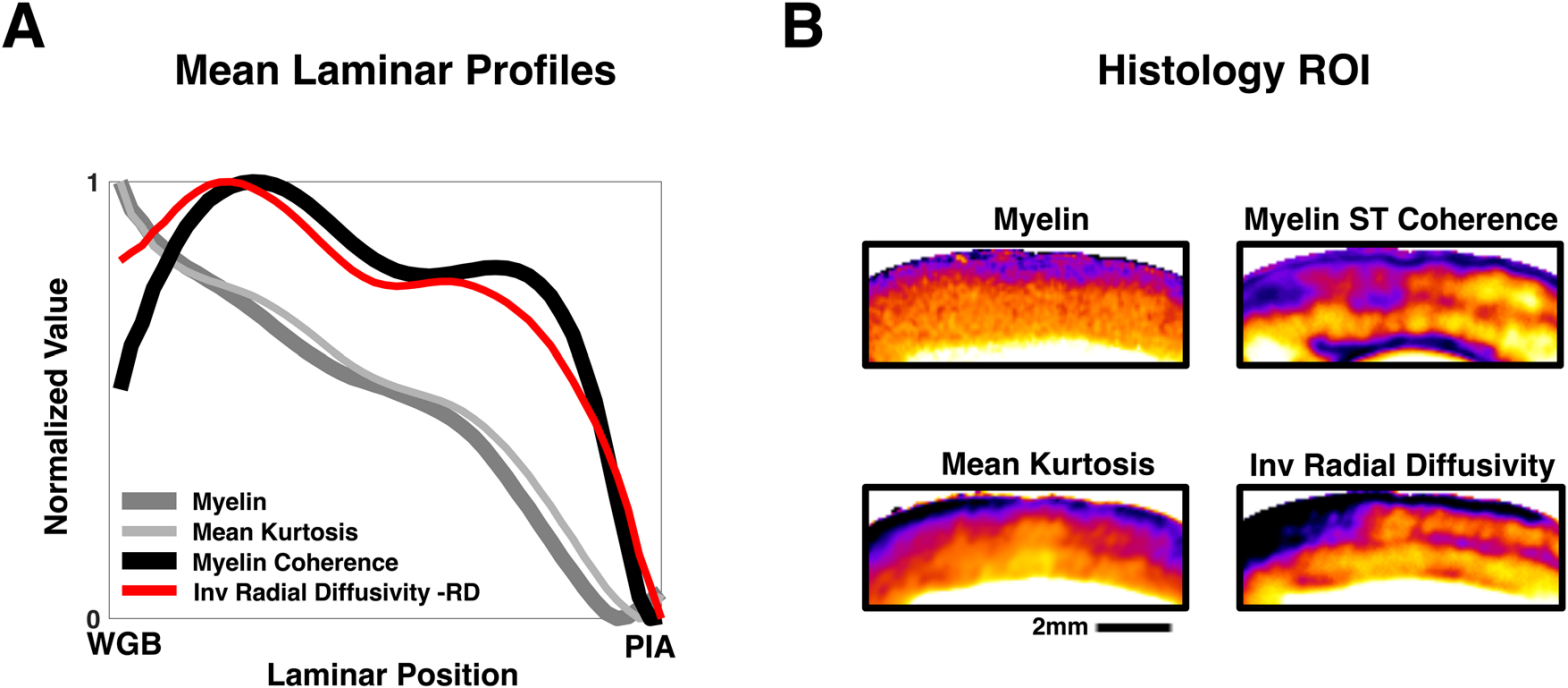
Differences in the relationships of RD and MK to histological myelin. (A) The mean laminar profiles (seeSection 2) of Histological myelin, Histological myelin coherence (seeSection 2) MK and -RD. MK closely matches the profile of histological myelin, while -RD more closely tracks myelin anisotropy. (B) Representative ROI of histological myelin, histological myelin coherence MK and -RD.

We found that the laminar profile of RD diverged significantly from that of myelin histology (Fig. 6A, B), but more closely matched that of myelin histological anisotropy. Although RD did show a correlation to myelin intensity, this correlation is notably weaker than MK’s, exhibiting a much wider spread of correlation coefficients between columnar parcels of cortex (Supplementary Fig. 3). We found that while the spatial pattern of RD was very sensitive to the directional content (Reveley et al., 2022) (Supplementary Fig. 3; Supplementary Text 1) of the myelinated axons as well as their density, it did not serve as a good quantitative measure of myelin content in the same way as mean kurtosis.

### 3.6 Mean diffusion kurtosis in area V1

**Fig. 7.**
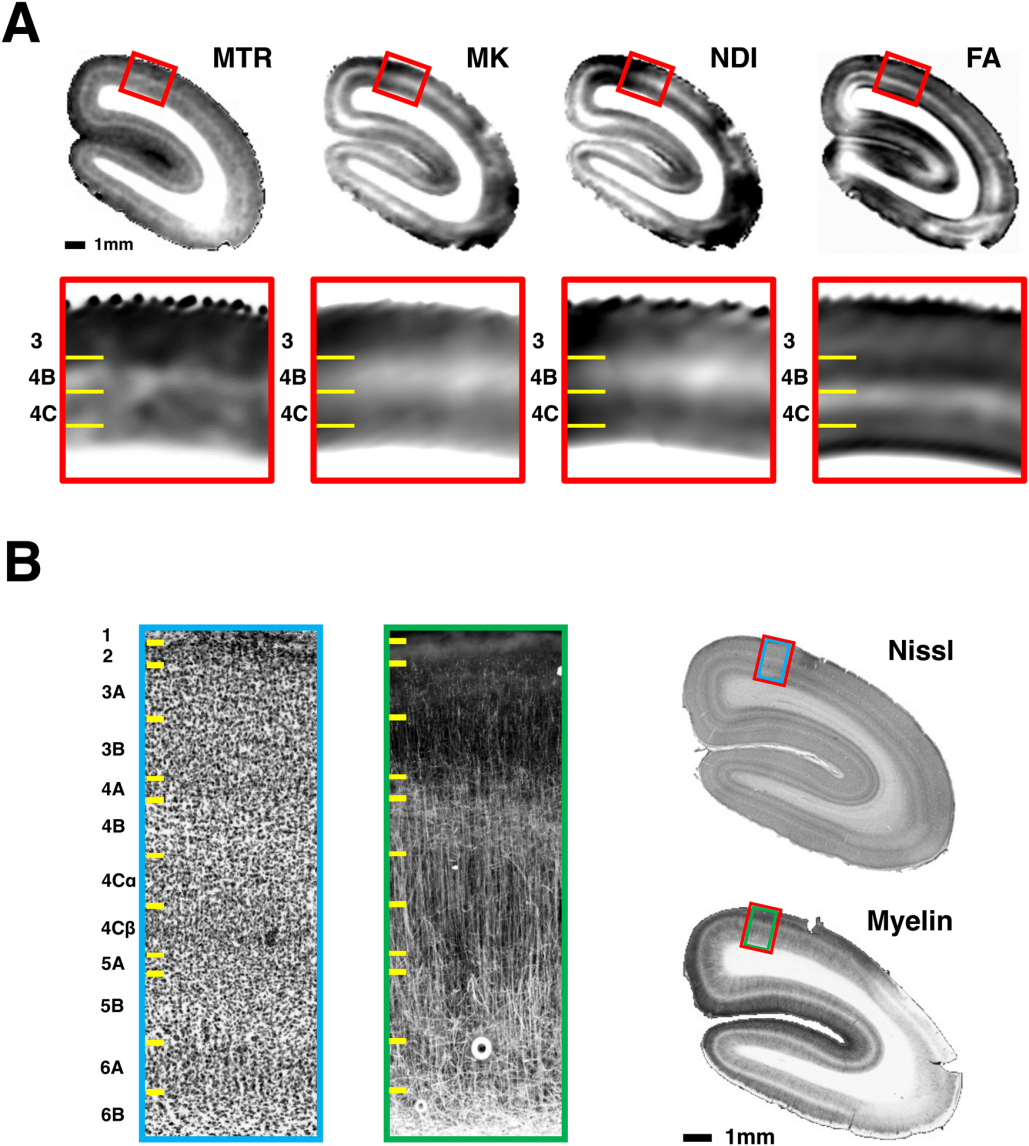
MTR, MK, and NDI reveal the same laminar properties of myelin in primary visual cortex, while FA appears to reflect non-myelinated components. (A) MTR, MK, NDI, and FA parameter maps expose different cortical layers and sublayers. In particular, the high myelin Gennari band of layer 4B is exposed as a high-intensity band of MTR, MK, and NDI, but a low-intensity band of FA. In contrast, Layer 4°C is shown as a low-intensity band of MTR, MK, and NDI but a high-intensity band of FA. (B) Nissl and myelin histology from the same subject matched to the MRI shown in A. See text for discussion.

There is wide regional variation in cytoarchitecture, myeloarchitecture, and neurite structure over the expanse of the cortical sheet. We were interested whether MK could assess the laminar structure of the primary visual cortex (V1), a well-studied cortical area with a unique laminar patterning of cell density and myelin (Balaram & Kaas, 2014;Casagrande & Kaas, 1994).Figure 7demonstrates the capacity of MTR, MK, NDI, and FA to reveal aspects of V1’s laminar architecture in the postmortem sample. Importantly, the laminae of highest intensity did not match among all the registered MRI measures. MTR, MK, and NDI matched well and showed their highest levels in layer 4B, the so-called Gennari band, reflecting its known high myelin content (Balaram & Kaas, 2014;Casagrande & Kaas, 1994) (Fig. 7A).

The abundant myelinated axons in 4B are visible in the corresponding histology to have a matted structure (Fig. 7B) and this lack of spatial coherence explains the low FA in this layer, and also in the deepest layer of cortex adjacent to the white matter. By contrast, layer 4°C was revealed by a high-intensity band of FA, which likely reflects an abundance of vertically oriented, non-myelinated neurites in this layer (Fig. 7A, right) (Reveley et al., 2022). Additionally, we found that radial diffusivity was relatively lower in layer 4°C where the myelinated axons are coherently organized, than in 4B where they are matted, despite the higher myelin content of the Gennari band in 4B (Supplementary Fig. 4; Supplementary Text 2). This likely reflects the sensitivity of RD to the organization of the myelinated fibers as shown inFigure 6B. Together, these results indicate that models based on multi-shelled diffusion acquisitions can distinguish and quantify the diffusion profiles of myelinated and non-myelinated neurites in the gray matter at a laminar level.

## 4 Discussion

In this study, we assessed the relationship between cortical myelin content and higher-order diffusion MRI model parameters based on multi-shelled acquisitions. We analyzed ultra-high-resolution dMRI and MTR scans of whole primate brains, as well as carefully co-registered histological data covering large areas of the frontal and parietal lobes. We examined the differences between cortical regions, as well as the laminar properties of the parameters within those regions. Our guiding hypothesis was that water restricted to the intra-axonal space by the myelin sheath would be reflected in a non-Gaussian molecule displacement profile, so we initially focused our investigation on mean kurtosis (MK). MK strongly correlated with MTR—a separate MRI contrast unrelated to diffusion, but closely linked to myelin levels—globally over the whole cortical sheet. Regions with the highest myelin content, and therefore the highest contrast-to-noise ratio, exhibited the strongest correlation with MK. Broadly similar results were obtained with the non-Gaussian (NG) parameter map obtained from MAP-MRI (Özarslan et al.,^2013^), and neurite density index (NDI) parameter map obtained from the NODDI biophysical model (Zhang et al., 2012). Each of these high-order diffusion measures closely tracked myelin, as well as one another. Specifically, they matched the monotonic decay of histological myelin levels from the white gray boundary to the pial surface, as well as the tangential variation across the cortical sheet. In the primary visual cortex, the MTR and MK measures captured the known distribution of myelin fibers in layer 4B. Of the three higher-order diffusion parameters we tested, NDI was unlike MK and NG since it does not explicitly reflect non-Gaussian diffusion processes. Potentially, both myelinated and unmyelinated neurites are intended to drive the NDI contrast (Zhang et al., 2012), but our analysis shows that NDI behaves similarly to MK, a measure of non-Gaussianity, in the environment of fixed primate gray matter.

The behavior of MK, NG, and NDI may be contrasted with the behavior of the Gaussian diffusion tensor in gray matter. Radial diffusivity (RD) exhibited sensitivity to the organization of the myelinated fibers as well as their density, rather than being attuned to myelin density alone. Fractional anisotropy (FA) showed only weak correspondence to myelin parameters, and in previous work we showed that FA is strongly driven by non-myelinated tissue components. Employing both the diffusion tensor and higher-order diffusion terms may offer a way to separate out the specific influences of myelinated and non-myelinated tissue components on the diffusion signal. Moreover, we found that MTR and MK were both myelin sensitive, despite obtaining their contrasts via different mechanisms. Thus, although they have correlated values in healthy tissue, MK and MTR might diverge for pathological cases in specific ways that have diagnostic value (Edwards et al., 2018;Kamiya et al., 2020).

### 4.1 Relation to previous findings

Our results cohere with and draw together several lines of converging evidence (Fukutomi et al., 2018;Guglielmetti et al., 2016;Jelescu et al., 2016;Kelm et al., 2016;Yoshida et al., 2013) at different spatial scales, to suggest that mean kurtosis has both sensitivity and specificity to myelin levels in healthy gray matter.

Mean kurtosis values have previously been shown to track changing myelination levels in vivo. For example,Guglielmetti et al. (2016)studied dMRI and immunohistochemical material from mice which had been treated with cuprizone, a drug that reversibly induces demyelination through toxicity to oligodendrocytes. The authors found that mean kurtosis decreased in the gray matter of motor and somatosensory cortex of the mouse following cuprizone administration and regained their values on recovery from the treatment. This was true except in areas of the cortex where the histology showed remyelination to be incomplete. Likewise, in a developmental study,Praet et al. (2018)assessed the correlation of mean kurtosis based on the optical density of co-registered myelin basic protein immunohistochemistry. They found that as myelin levels in the motor cortex increased during development, cortical MK tended to increase, and FA tended to decrease. These rodent studies emphasized longitudinal changes in experimental cohorts, rather than detail within the cortex of particular subjects.

A few studies have examined the distribution of higher-order diffusion metrics in groups of human subjects at clinical resolutions over the whole brain.Bester et al. (2015)examined diffusion kurtosis in a cohort of subjects suffering from multiple sclerosis (MS), a demyelinating disease. They found that MK was significantly lower in the gray matter of MS patients compared to controls, and that MK was inversely correlated with executive function where poor function presumably reflects cortical demyelination.Fukutomi et al. (2018)found that NDI was strongly correlated to myelin density as estimated by the T1w/T2w ratio, in a group-based cortical surface-based study of 505 healthy subjects from the human connectome project. These studies both suggest that the present findings in an ex-vivo primate model are strongly relevant to human cortical tissue scanned in-vivo, at much lower spatial resolutions.

Zhu et al. (2021)compared MK in a cohort of six postmortem macaque brains at lower spatial resolutions of around 600 µm in plane, gathered in 2 mm slices, to publicly available neurofilament (SMI-32) immunohistochemistry from a different animal. They found qualitative correspondence between the two measures over the cortex as a whole; however, they were not able to examine the histology/dMRI relationship at a fine spatial scale in the same brains. SMI-32 is reactive in a subset of large pyramidal cells in the cortical gray matter and is closely associated with myelination (Kirkcaldie et al., 2002), because those same cells give rise to the largest myelinated axons in cortex. The broad correspondenceZhu et al. (2021)noted at comparatively low spatial resolution may be a proxy for average cortical myelin levels, rather than indicating a direct link between MK and the neurofilaments themselves. More detailed work is required to address this question.

A handful of studies (Lazari & Lipp, 2021) have examined the relationship of diffusion kurtosis to the detailed properties of myelinated axons in murine white matter using electron microscopy.Kelm et al. (2016)studied the relation of myelin to diffusion kurtosis in an ex-vivo cohort of knockout and control mice with varying degrees of hypomyelination. They found that MK correlated to the fraction of myelin as estimated from the electron microscopy data in four white matter ROI across their subject pool.Jelescu et al. (2016)also found that MK exhibited sensitivity to cuprizone-induced demyelination in an analysis of electron microscopy in the mouse corpus callosum.

### 4.2 Caveats and limitations of the present study

Connecting MRI data to underlying tissue parameters requires certain assumptions, so it is important to identify where such assumptions might break down. Importantly, while the focus of the present study was on myelin, both MTR and MK may also have sensitivity to other tissue properties. For example, MTR is known to vary with water content, as brought about by edema, inflammation, and other immune responses (Heath et al., 2018;Vavasour et al., 2011). Likewise, beyond marking axonal myelination, MK varies with other factors that increase tissue “complexity”, such as gliosis or gliomas (Wu & Cheung, 2010). These caveats may be particularly important in the translation of the ex-vivo data presented here to the living brain. In this study, measures of diffusion kurtosis or non-Gaussian diffusion were found to correlate to the tissue myelin content. We presume this is due to restricted water diffusion in the myelinated axons, but it is worth mentioning that the relationship is empirical and has limitations in theory. For example, in a given region of brain, there could be more than one way for increased myelin content to occur—thicker myelin on the same number of axons or more myelinated axons per volume. Our results do not demonstrate whether MK can distinguish these scenarios; with that said, we did find empirically that MK closely tracked both MTR and histological myelin density in the gray matter, which contains axons of a wide variety of calibers. Isolating any myelin-unique component of MRI signals is particularly difficult in the cortical gray matter, because multiple tissue parameters correlate spatially with one another. For example, in the cortex, the largest myelinated and non-myelinated neurites both have a strong vertical orientation bias and are spatially enmeshed (Buxhoeveden & Casanova, 2002;Peters et al., 1997;Peters & Sethares, 1996). Nonetheless, our demonstration of a laminar gradient of MK in the cortex does suggest that myelin is the determining influence, since this is a signature of cortical myelin levels (Braitenberg, 1962), whereas the density of unmyelinated neurites is typically high in superficial cortical layers, near the pia (Peters, 2010;Peters et al., 1997).

Diffusion parameters are sensitive to the state of the tissue. In the present study, we exclusively addressed fixed, ex-vivo tissue scanned at diffusion times in the range 10–20 ms (seeSection 2). Acquisition in living tissue might give contrasting results, since both diffusivities and higher-order diffusion measures differ in living tissue. Likewise, while the MTR method is suitable for ex-vivo studies, where T1 times are equalized by Gd doping, T1-weighted imaging is likely more useful for in-vivo studies, due to its higher contrast-to-noise ratio and lower specific absorption rate (SAR) exposure to patients. We note thatFukutomi et al. (2018)found good agreement between NODDI NDI and myelin as estimated by the T1w/T2w ratio over the whole human brain in-vivo; so far, the ex-vivo and in-vivo findings are supportive to each other. Work to assess these issues and develop sophisticated, empirically grounded models of complex diffusion processes in gray matter is ongoing (Jelescu et al., 2022;Lee et al., 2020).

## Supplementary Material

Supplementary Material

## Data Availability

The data employed here were used in two previous studies (Liu et al., 2018,^2020^;Reveley et al., 2022).

## References

[b1] Andica , C. , Kamagata , K. , Hatano , T. , Saito , Y. , Ogaki , K. , Hattori , N. , & Aoki , S. ( 2020 ). MR biomarkers of degenerative brain disorders derived from diffusion imaging . Journal of Magnetic Resonance Imaging , 52 ( 6 ), 1620 – 1636 . 10.1002/jmri.27019 31837086 PMC7754336

[b2] Avants , B. B. , Tustison , N. J. , Song , G. , Cook , P. A. , Klein , A. , & Gee , J. C. ( 2011 ). A reproducible evaluation of ANTs similarity metric performance in brain image registration . NeuroImage , 54 ( 3 ), 2033 – 2044 . 10.1016/j.neuroimage.2010.09.025 20851191 PMC3065962

[b3] Avram , A. V. , Saleem , K. S. , Komlosh , M. E. , Yen , C. C. , Ye , F. Q. , & Basser , P. J. ( 2022 ). High-resolution cortical MAP-MRI reveals areal borders and laminar substructures observed with histological staining: High-resolution cortical MAP-MRI . NeuroImage , 264 , 119653 . 10.1016/j.neuroimage.2022.119653 36257490

[b4] Balaram , P. , & Kaas , J. H. ( 2014 ). Towards a unified scheme of cortical lamination for primary visual cortex across primates: Insights from NeuN and VGLUT2 immunoreactivity . Frontiers in Neuroanatomy , 8 , 81 . 10.3389/fnana.2014.00081 25177277 PMC4133926

[b5] Beaulieu , C. ( 2002 ). The basis of anisotropic water diffusion in the nervous system—A technical review . NMR in Biomedicine , 15 ( 7–8 ), 435 – 455 . 10.1002/nbm.782 12489094

[b6] Behrens , T. , Berg , H. J. , Jbabdi , S. , Rushworth , M. , & Woolrich , M. ( 2007 ). Probabilistic diffusion tractography with multiple fibre orientations: What can we gain? Neuroimage , 34 ( 1 ), 144 – 155 . 10.1016/j.neuroimage.2006.09.018 17070705 PMC7116582

[b7] Bester , M. , Jensen , J. H. , Babb , J. S. , Tabesh , A. , Miles , L. , Herbert , J. , Grossman , R. I. , & Inglese , M. ( 2015 ). Non-Gaussian diffusion MRI of gray matter is associated with cognitive impairment in multiple sclerosis . Multiple Sclerosis , 21 ( 7 ), 935 – 944 . 10.1177/1352458514556295 25392318 PMC4429046

[b8] Bock , N. A. , Hashim , E. , Kocharyan , A. , & Silva , A. C. ( 2011 ). Visualizing myeloarchitecture with magnetic resonance imaging in primates . Annals of the New York Academy of Sciences , 1225 ( S1 ), E171 – E181 . 10.1111/j.1749-6632.2011.06000.x 21599695 PMC5027904

[b9] Braitenberg , V. ( 1962 ). A note on myeloarchitectonics . Journal of Comparative Neurology , 118 ( 2 ), 141 – 156 . 10.1002/cne.901180202 13872421

[b10] Budde , M. D. , & Frank , J. A. ( 2012 ). Examining brain microstructure using structure tensor analysis of histological sections . NeuroImage , 63 ( 1 ), 1 – 10 . 10.1016/j.neuroimage.2012.06.042 22759994

[b11] Buxhoeveden , D. P. , & Casanova , M. F. ( 2002 ). The minicolumn hypothesis in neuroscience . Brain , 125 ( 5 ), 935 – 951 . 10.1093/brain/awf110 11960884

[b12] Casagrande , V. A. , & Kaas , J. H. ( 1994 ). The afferent, intrinsic, and efferent connections of primary visual cortex in primates . In A. Peters & K. S. Rockland (Eds.), Primary Visual Cortex in Primates. Cerebral Cortex Vol. 10 , pp. 201 – 259 . Springer . 10.1007/978-1-4757-9628-5_5

[b13] Daducci , A. , Canales-Rodríguez , E. J. , Zhang , H. , Dyrby , T. B. , Alexander , D. C. , & Thiran , J. P. ( 2015 ). Accelerated Microstructure Imaging via Convex Optimization (AMICO) from diffusion MRI data . NeuroImage , 105 , 32 – 44 . 10.1016/j.neuroimage.2014.10.026 25462697

[b14] Edwards , L. J. , Kirilina , E. , Mohammadi , S. , & Weiskopf , N. ( 2018 ). Microstructural imaging of human neocortex in vivo . NeuroImage , 182 , 184 – 206 . 10.1016/j.neuroimage.2018.02.055 29588229

[b15] Falangola , M. F. , Guilfoyle , D. N. , Tabesh , A. , Hui , E. S. , Nie , X. , Jensen , J. H. , Gerum , S. V. , Hu , C. , Lafrancois , J. , Collins , H. R. , & Helpern , J. A. ( 2014 ). Histological correlation of diffusional kurtosis and white matter modeling metrics in cuprizone-induced corpus callosum demyelination . NMR in Biomedicine , 27 ( 8 ), 948 – 957 . 10.1002/nbm.3140 24890981 PMC5297373

[b16] Fukutomi , H. , Glasser , M. F. , Zhang , H. , Autio , J. A. , Coalson , T. S. , Okada , T. , Togashi , K. , Van Essen , D. C. , & Hayashi , T. ( 2018 ). Neurite imaging reveals microstructural variations in human cerebral cortical gray matter . NeuroImage , 182 , 488 – 499 . 10.1016/j.neuroimage.2018.02.017 29448073 PMC6326835

[b17] Gallyas , F. ( 1979 ). Silver staining of myelin by means of physical development . Neurological Research , 1 ( 2 ), 203 – 209 . 10.1080/01616412.1979.11739553 95356

[b18] Glasser , M. F. , Goyal , M. S. , Preuss , T. M. , Raichle , M. E. , & Van Essen , D. C . ( 2014 ). Trends and properties of human cerebral cortex: Correlations with cortical myelin content . NeuroImage , 93 , 165 – 175 . 10.1016/j.neuroimage.2013.03.060 23567887 PMC3795824

[b19] Grossman , R. I. , Gomori , J. M. , Ramer , K. N. , Lexa , F. J. , & Schnall , M. D. ( 1994 ). Magnetization transfer: Theory and clinical applications in neuroradiology . Radiographics , 14 ( 2 ), 279 – 290 . 10.1148/radiographics.14.2.8190954 8190954

[b20] Guglielmetti , C. , Veraart , J. , Roelant , E. , Mai , Z. , Daans , J. , Van Audekerke , J. , Naeyaert , M. , Vanhoutte , G. , Delgado y Palacios , R. , Praet , J. , Fieremans , E. , Ponsaerts , P. , Sijbers , J. , Van der Linden , A. , & Verhoye , M. ( 2016 ). Diffusion kurtosis imaging probes cortical alterations and white matter pathology following cuprizone induced demyelination and spontaneous remyelination . NeuroImage , 125 , 363 – 377 . 10.1016/j.neuroimage.2015.10.052 26525654 PMC4935929

[b21] Heath , F. , Hurley , S. A. , Johansen-Berg , H. , & Sampaio-Baptista , C. ( 2018 ). Advances in noninvasive myelin imaging . Developmental Neurobiology , 78 ( 2 ), 136 – 151 . 10.1002/dneu.22552 29082667 PMC5813152

[b22] Heinrich , M. P. , Jenkinson , M. , Bhushan , M. , Matin , T. , Gleeson , F. V. , Brady , S. M. , & Schnabel , J. A. ( 2012 ). MIND: Modality independent neighbourhood descriptor for multi-modal deformable registration . Medical Image Analysis , 16 ( 7 ), 1423 – 1435 . 10.1016/j.media.2012.05.008 22722056

[b23] Henkelman , R. M. , Stanisz , G. J. , & Graham , S. J. ( 2001 ). Magnetization transfer in MRI: A review . NMR in Biomedicine , 14 ( 2 ), 57 – 64 . 10.1002/nbm.683 11320533

[b24] Henriques , R. N. , Jespersen , S. N. , Jones , D. K. , & Veraart , J. ( 2021 ). Toward more robust and reproducible diffusion kurtosis imaging . Magnetic Resonance in Medicine , 86 ( 3 ), 1600 – 1613 . 10.1002/mrm.28730 33829542 PMC8199974

[b25] Howard , A. F. , Huszar , I. N. , Smart , A. , Cottaar , M. , Daubney , G. , Hanayik , T. , Khrapitchev , A. A. , Mars , R. B. , Mollink , J. , Scott , C. , Sibson , N. R. , Sallet , J. , Jbabdi , S. , & Miller , K. L. ( 2022 ). An open resource combining multi-contrast MRI and microscopy in the macaque brain . Nature Communications , 14 , 4320 . 10.1038/s41467-023-39916-1 PMC10356772 37468455

[b26] Jelescu , I. O. , de Skowronski , A. , Geffroy , F. , Palombo , M. , & Novikov , D. S. ( 2022 ). Neurite Exchange Imaging ((NEXI): A minimal model of diffusion in gray matter with inter-compartment water exchange . NeuroImage , 256 , 119277 . 10.1016/j.neuroimage.2022.119277 35523369 PMC10363376

[b27] Jelescu , I. O. , Palombo , M. , Bagnato , F. , & Schilling , K. G. ( 2020 ). Challenges for biophysical modeling of microstructure . Journal of Neuroscience Methods , 344 , 108861 . 10.1016/j.jneumeth.2020.108861 32692999 PMC10163379

[b28] Jelescu , I. O. , Zurek , M. , Winters , K. V. , Veraart , J. , Rajaratnam , A. , Kim , N. S. , Babb , J. S. , Shepherd , T. M. , Novikov , D. S. , Kim , S. G. , & Fieremans , E. ( 2016 ). In vivo quantification of demyelination and recovery using compartment-specific diffusion MRI metrics validated by electron microscopy . NeuroImage , 132 , 104 – 114 . 10.1016/j.neuroimage.2016.02.004 26876473 PMC4851889

[b29] Jensen , J. H. , Helpern , J. A. , Ramani , A. , Lu , H. , & Kaczynski , K. ( 2005 ). Diffusional kurtosis imaging: The quantification of non-Gaussian water diffusion by means of magnetic resonance imaging . Magnetic Resonance in Medicine , 53 ( 6 ), 1432 – 1440 . 10.1002/mrm.20508 15906300

[b30] Jespersen , S. N. , Bjarkam , C. R. , Nyengaard , J. R. , Chakravarty , M. M. , Hansen , B. , Vosegaard , T. , Østergaard , L. , Yablonskiy , D. , Nielsen , N. C. , & Vestergaard-Poulsen , P. ( 2010 ). Neurite density from magnetic resonance diffusion measurements at ultrahigh field: Comparison with light microscopy and electron microscopy . NeuroImage , 49 ( 1 ), 205 – 216 . 10.1016/j.neuroimage.2009.08.053 19732836 PMC2862296

[b31] Jespersen , S. N. , Kroenke , C. D. , Østergaard , L. , Ackerman , J. J. H. , & Yablonskiy , D. A. ( 2007 ). Modeling dendrite density from magnetic resonance diffusion measurements . NeuroImage , 34 ( 4 ), 1473 – 1486 . 10.1016/j.neuroimage.2006.10.037 17188901

[b32] Jespersen , S. N. , Leigland , L. A. , Cornea , A. , & Kroenke , C. D. ( 2012 ). Determination of axonal and dendritic orientation distributions within the developing cerebral cortex by diffusion tensor imaging . IEEE Transactions on Medical Imaging , 31 ( 1 ), 16 – 32 . 10.1109/TMI.2011.2162099 21768045 PMC3271123

[b33] Jito , J. , Nakasu , S. , Ito , R. , Fukami , T. , Morikawa , S. , & Inubushi , T. ( 2008 ). Maturational changes in diffusion anisotropy in the rat corpus callosum: Comparison with quantitative histological evaluation . Journal of Magnetic Resonance Imaging , 28 ( 4 ), 847 – 854 . 10.1002/jmri.21496 18821626

[b34] Johansen-Berg , H. , & Behrens , T. E. J. (Eds.) ( 2013 ). Diffusion MRI: From quantitative measurement to in vivo neuroanatomy . Academic Press . https://shop.elsevier.com/books/diffusion-mri/johansen-berg/978-0-12-396460-1

[b35] Kamiya , K. , Hori , M. , & Aoki , S. ( 2020 ). NODDI in clinical research . Journal of Neuroscience Methods , 346 , 108908 . 10.1016/j.jneumeth.2020.108908 32814118

[b36] Kandel , E. R. , Schwartz , J. H. , Jessell , T. M. , Siegelbaum , S. A. , & Hudspeth , A. J. ( 2013 ). Principles of Neural Science , 5th ed. McGraw Hill Professional . ISBN: 978-0-07-139011-8. https://accessbiomedicalscience.mhmedical.com/book.aspx?bookid=1049

[b37] Kelm , N. D. , West , K. L. , Carson , R. P. , Gochberg , D. F. , Ess , K. C. , & Does , M. D. ( 2016 ). Evaluation of diffusion kurtosis imaging in ex vivo hypomyelinated mouse brains . NeuroImage , 124 , 612 – 626 . 10.1016/j.neuroimage.2015.09.028 26400013 PMC4651761

[b38] Kirkcaldie , M. T. K. , Dickson , T. C. , King , C. E. , Grasby , D. , Riederer , B. M. , & Vickers , J. C. ( 2002 ). Neurofilament triplet proteins are restricted to a subset of neurons in the rat neocortex . Journal of Chemical Neuroanatomy , 24 ( 3 ), 163 – 171 . 10.1016/S0891-0618(02)00043-1 12297262

[b39] Koenig , S. H. ( 1991 ). Cholesterol of myelin is the determinant of gray‐white contrast in MRI of brain . Magnetic Resonance in Medicine , 20 ( 2 ), 285 – 291 . 10.1002/mrm.1910200210 1775053

[b40] Lazari , A. , & Lipp , I. ( 2021 ). Can MRI measure myelin? Systematic review, qualitative assessment, and meta-analysis of studies validating microstructural imaging with myelin histology . NeuroImage , 230 , 117744 . 10.1016/j.neuroimage.2021.117744 33524576 PMC8063174

[b41] Lee , H. H. , Papaioannou , A. , Novikov , D. S. , & Fieremans , E. ( 2020 ). In vivo observation and biophysical interpretation of time-dependent diffusion in human cortical gray matter . NeuroImage , 222 , 117054 . 10.1016/j.neuroimage.2020.117054 32585341 PMC7736473

[b42] Lewis , J. W. , & Van Essen , D. C . ( 2000 ). Mapping of architectonic subdivisions in the macaque monkey, with emphasis on parieto-occipital cortex . The Journal of Comparative Neurology , 428 ( 1 ), 79 – 111 . 10.1002/1096-9861(20001204)428:1<79::AID-CNE7>3.0.CO;2-Q 11058226

[b43] Liu , C. , Ye , F. Q. , Newman , J. D. , Szczupak , D. , Tian , X. , Yen , C. C. C. , Majka , P. , Glen , D. , Rosa , M. G. P. , Leopold , D. A. , & Silva , A. C. ( 2020 ). A resource for the detailed 3D mapping of white matter pathways in the marmoset brain . Nature Neuroscience , 23 ( 2 ), 271 – 280 . 10.1038/s41593-019-0575-0 31932765 PMC7007400

[b44] Liu , C. , Ye , F. Q. , Yen , C. C.-C. , Newman , J. D. , Glen , D. , Leopold , D. A. , & Silva , A. C. ( 2018 ). A digital 3D atlas of the marmoset brain based on multi-modal MRI . NeuroImage , 169 , 106 – 116 . 10.1016/j.neuroimage.2017.12.004 29208569 PMC5856608

[b45] Mancini , M. , Karakuzu , A. , Cohen-Adad , J. , Cercignani , M. , Nichols , T. E. , & Stikov , N. ( 2020 ). An interactive meta-analysis of MRI biomarkers of Myelin . eLife , 9 , 1 – 23 . 10.7554/eLife.61523 PMC7647401 33084576

[b46] McKavanagh , R. , Torso , M. , Jenkinson , M. , Kolasinski , J. , Stagg , C. J. , Esiri , M. M. , McNab , J. A. , Johansen-Berg , H. , Miller , K. L. , & Chance , S. A. ( 2019 ). Relating diffusion tensor imaging measurements to microstructural quantities in the cerebral cortex in multiple sclerosis . Human Brain Mapping , 40 ( 15 ), 4417 – 4431 . 10.1002/hbm.24711 31355989 PMC6772025

[b47] McNab , J. A. , Polimeni , J. R. , Wang , R. , Augustinack , J. C. , Fujimoto , K. , Stevens , A. , Janssens , T. , Farivar , R. , Folkerth , R. D. , Vanduffel , W. , & Wald , L. L. ( 2013 ). Surface based analysis of diffusion orientation for identifying architectonic domains in the in vivo human cortex . NeuroImage , 69 , 87 – 100 . 10.1016/j.neuroimage.2012.11.065 23247190 PMC3557597

[b48] Morell , P. , & Quarles , R. H. ( 1999 ). Myelin formation, structure and biochemistry . In Siegel , G. J. , Agranoff , B. W. , Albers , R. W. , et al. (Eds.). Basic neurochemistry: Molecular, cellular and medical aspects . 6th edition. Philadelphia : Lippincott-Raven ; Chapter 4. https://www.ncbi.nlm.nih.gov/books/NBK20402/

[b49] Novikov , D. S. , Fieremans , E. , Jespersen , S. N. , & Kiselev , V. G. ( 2019 ). Quantifying brain microstructure with diffusion MRI: Theory and parameter estimation . NMR in Biomedicine , 32 ( 4 ), 1 – 53 . 10.1002/nbm.3998 PMC6481929 30321478

[b50] Ono , J. , Harada , K. , Takahashi , M. , Maeda , M. , Sakurai , K. , Sakai , N. , Kagawa , T. , Fritz-Zieroth , B. , Nagai , T. , Nihei , A. , Hashimoto , S. , & Okada , S. ( 1995 ). Differentiation between dysmyelination and demyelination using magnetic resonance diffusional anisotropy . Brain Research , 671 , 141 – 148 . 10.1016/0006-8993(94)01335-F 7728526

[b51] Ouyang , M. , Jeon , T. , Sotiras , A. , Peng , Q. , Mishra , V. , Halovanic , C. , Chen , M. , Chalak , L. , Rollins , N. , Roberts , T. P. L. , Davatzikos , C. , & Huang , H. ( 2019 ). Differential cortical microstructural maturation in the preterm human brain with diffusion kurtosis and tensor imaging . Proceedings of the National Academy of Sciences of the United States of America , 116 ( 10 ), 4681 – 4688 . 10.1073/pnas.1812156116 30782802 PMC6410816

[b52] Özarslan , E. , Koay , C. G. , Shepherd , T. M. , Komlosh , M. E. , İrfanoğlu , M. O. , Pierpaoli , C. , Basser , P. J. , Irfanoǧlu , M. O. , Pierpaoli , C. , Basser , P. J. , İrfanoğlu , M. O. , Pierpaoli , C. , & Basser , P. J. ( 2013 ). Mean apparent propagator (MAP) MRI: A novel diffusion imaging method for mapping tissue microstructure . NeuroImage , 78 , 16 – 32 . 10.1016/j.neuroimage.2013.04.016 23587694 PMC4059870

[b53] Paxinos , G. , Watson , C. , Petrides , M. , Rosa , M. , & Tokuno , H. ( 2013 ). The marmoset brain in stereotaxic coordinates . Elsevier Academic Press . https://espace.curtin.edu.au/handle/20.500.11937/40725

[b54] Paydar , A. , Fieremans , E. , Nwankwo , J. I. , Lazar , M. , Sheth , H. D. , Adisetiyo , V. , Helpern , J. A. , Jensen , J. H. , & Milla , S. S. ( 2014 ). Diffusional kurtosis imaging of the developing brain . American Journal of Neuroradiology , 35 ( 4 ), 808 – 814 . 10.3174/ajnr.A3764 24231848 PMC7965814

[b55] Peters , A. ( 2010 ). The morphology of minicolumns . In G. J. Blatt (Ed.), The Neurochemical Basis of Autism: From Molecules to Minicolumns (pp. 1 – 295 ). Springer . 10.1007/978-1-4419-1272-5

[b56] Peters , A. , Cifuentes Manuel , J., & Sethares , C. ( 1997 ). The organization of pyramidal cells in area 18 of the rhesus monkey . Cerebral Cortex , 7 ( 5 ), 405 – 421 . 10.1093/cercor/7.5.405 9261571

[b57] Peters , A. , & Sethares , C. ( 1996 ). Myelinated axons and the pyramidal cell modules in monkey primary visual cortex . Journal of Comparative Neurology , 365 ( 2 ), 232 – 255 . 10.1002/(SICI)1096-9861(19960205)365:2<232::AID-CNE3>3.0.CO;2-6 8822167

[b58] Pierpaoli , C. , Walker , L. , Irfanoglu , M. , Barnett , A. , Basser , P. , Chang , L. , Koay , C. , Pajevic , S. , Rohde , G. , Sarlls , J. , & Wu , M. ( 2010 ). TORTOISE: An integrated software package for processing of diffusion MRI data . In ISMRM 18th annual meeting (Vol. 1597 ). Germany. https://archive.ismrm.org/2010/1597.html

[b59] Praet , J. , Manyakov , N. V. , Muchene , L. , Mai , Z. , Terzopoulos , V. , De Backer , S. , Torremans , A. , Guns , P. J. , Van De Casteele , T. , Bottelbergs , A. , Van Broeck , B. , Sijbers , J. , Smeets , D. , Shkedy , Z. , Bijnens , L. , Pemberton , D. J. , Schmidt , M. E. , Van Der Linden , A. , & Verhoye , M. ( 2018 ). Diffusion kurtosis imaging allows the early detection and longitudinal follow-up of amyloid-β-induced pathology . Alzheimer’s Research and Therapy , 10 ( 1 ), 1 . 10.1186/s13195-017-0329-8 PMC6389136 29370870

[b60] Reveley , C. , Seth , A. K. , Pierpaoli , C. , Silva , A. C. , Yu , D. , Saunders , R. C. , Leopold , D. A. , & Ye , F. Q. ( 2015 ). Superficial white matter fiber systems impede detection of long-range cortical connections in diffusion MR tractography . Proceedings of the National Academy of Sciences of the United States of America , 112 ( 21 ), E2820 – E2828 . 10.1073/pnas.1418198112 25964365 PMC4450402

[b61] Reveley , C. , Ye , F. Q. , Mars , R. B. , Matrov , D. , Chudasama , Y. , & Leopold , D. A. ( 2022 ). Diffusion MRI anisotropy in the cerebral cortex is determined by unmyelinated tissue features . Nature Communications , 13 ( 1 ), 6702 . 10.1038/s41467-022-34328-z PMC9637141 36335105

[b62] Rockland , K. S. , & Ichinohe , N. ( 2004 ). Some thoughts on cortical minicolumns . Experimental Brain Research , 158 ( 3 ), 265 – 277 . 10.1007/s00221-004-2024-9 15365664

[b63] Schindelin , J. , Arganda-Carreras , I. , Frise , E. , Kaynig , V. , Longair , M. , Pietzsch , T. , Preibisch , S. , Rueden , C. , Saalfeld , S. , Schmid , B. , Tinevez , J. Y. , White , D. J. , Hartenstein , V. , Eliceiri , K. , Tomancak , P. , & Cardona , A. ( 2012 ). Fiji: An open-source platform for biological-image analysis . Nature Methods , 9 ( 7 ), 676 – 682 . 10.1038/nmeth.2019 22743772 PMC3855844

[b64] Schüz , A. , & Braitenberg , V. ( 2002 ). The human cortical white matter: Quantitative aspects of cortico-cortical long-range connectivity . In Schüz , A. , & Robert , M. (Eds.). Cortical areas: Unity and diversity . CRC press. 10.1201/9780203299296

[b65] Song , S. K. , Sun , S. W. , Ju , W. K. , Lin , S. J. , Cross , A. H. , & Neufeld , A. H. ( 2003 ). Diffusion tensor imaging detects and differentiates axon and myelin degeneration in mouse optic nerve after retinal ischemia . NeuroImage , 20 ( 3 ), 1714 – 1722 . 10.1016/j.neuroimage.2003.07.005 14642481

[b66] Song , S. K. , Sun , S. W. , Ramsbottom , M. J. , Chang , C. , Russell , J. , & Cross , A. H. ( 2002 ). Dysmyelination revealed through MRI as increased radial (but unchanged axial) diffusion of water . NeuroImage , 17 ( 3 ), 1429 – 1436 . 10.1006/nimg.2002.1267 12414282

[b67] Song , S. K. , Yoshino , J. , Le , T. Q. , Lin , S. J. , Sun , S. W. , Cross , A. H. , & Armstrong , R. C. ( 2005 ). Demyelination increases radial diffusivity in corpus callosum of mouse brain . NeuroImage , 26 ( 1 ), 132 – 140 . 10.1016/j.neuroimage.2005.01.028 15862213

[b68] Stikov , N. , Perry , L. M. , Mezer , A. , Rykhlevskaia , E. , Wandell , B. A. , Pauly , J. M. , & Dougherty , R. F. ( 2011 ). Bound pool fractions complement diffusion measures to describe white matter micro and macrostructure . NeuroImage , 54 ( 2 ), 1112 – 1121 . 10.1016/j.neuroimage.2010.08.068 20828622 PMC2997845

[b69] Torso , M. , Bozzali , M. , Zamboni , G. , Jenkinson , M. , & Chance , S. A. ( 2021 ). Detection of Alzheimer’s disease using cortical diffusion tensor imaging . Human Brain Mapping , 42 ( 4 ), 967 – 977 . 10.1002/hbm.25271 33174658 PMC7856641

[b70] Underhill , H. R. , Yuan , C. , & Yarnykh , V. L. ( 2009 ). Direct quantitative comparison between cross-relaxation imaging and diffusion tensor imaging of the human brain at 3.0 T . NeuroImage , 47 ( 4 ), 1568 – 1578 . 10.1016/j.neuroimage.2009.05.075 19500678

[b71] Vavasour , I. M. , Laule , C. , Li , D. K. B. , Traboulsee , A. L. , & MacKay , A. L. ( 2011 ). Is the magnetization transfer ratio a marker for myelin in multiple sclerosis? Journal of Magnetic Resonance Imaging , 33 ( 3 ), 710 – 718 . 10.1002/jmri.22441 21563257

[b72] Vogt , C. , & Vogt , O. ( 1919 ). Allgemeinere Ergebnisse unserer Hirnforschung . Journal für Psychologie und Neurologie , 25 , 279 – 461 . http://ci.nii.ac.jp/naid/10024136586/en/

[b73] Wheeler-Kingshott , C. A. M. , & Cercignani , M. ( 2009 ). About ‘axial’ and ‘radial’ diffusivities . Magnetic Resonance in Medicine , 61 ( 5 ), 1255 – 1260 . 10.1002/mrm.21965 19253405

[b74] Wolff , S. D. , & Balaban , R. S. ( 1989 ). Magnetization transfer contrast (MTC) and tissue water proton relaxation in vivo . Magnetic Resonance in Medicine , 10 ( 1 ), 135 – 144 . 10.1002/mrm.1910100113 2547135

[b75] Woolrich , M. W. , Jbabdi , S. , Patenaude , B. , Chappell , M. , Makni , S. , Behrens , T. , Beckmann , C. , Jenkinson , M. , & Smith , S. M. ( 2009 ). Bayesian analysis of neuroimaging data in FSL . NeuroImage , 45 ( 1, Supp. 1 ), S173 – S186 . 10.1016/j.neuroimage.2008.10.055 19059349

[b76] Wu , E. X. , & Cheung , M. M. ( 2010 ). MR diffusion kurtosis imaging for neural tissue characterization . NMR in Biomedicine , 23 ( 7 ), 836 – 848 . 10.1002/nbm.1506 20623793

[b77] Yoshida , M. , Hori , M. , Yokoyama , K. , Fukunaga , I. , Suzuki , M. , Kamagata , K. , Shimoji , K. , Nakanishi , A. , Hattori , N. , Masutani , Y. , & Aoki , S. ( 2013 ). Diffusional kurtosis imaging of normal-appearing white matter in multiple sclerosis: Preliminary clinical experience . Japanese Journal of Radiology , 31 ( 1 ), 50 – 55 . 10.1007/s11604-012-0147-7 23086313

[b78] Zhang , H. , Schneider , T. , Wheeler-Kingshott , C. A. , & Alexander , D. C. ( 2012 ). NODDI: Practical in vivo neurite orientation dispersion and density imaging of the human brain . NeuroImage , 61 ( 4 ), 1000 – 1016 . 10.1016/j.neuroimage.2012.03.072 22484410

[b79] Zhu , T. , Peng , Q. , Ouyang , A. , & Huang , H. ( 2021 ). Neuroanatomical underpinning of diffusion kurtosis measurements in the cerebral cortex of healthy macaque brains . Magnetic Resonance in Medicine , 85 ( 4 ), 1895 – 1908 . 10.1002/mrm.28548 33058286 PMC8934732

[b80] Zhuo , J. , & Gullapalli , R. P. ( 2020 ). Diffusion kurtosis imaging . In M. Mannil & S. F.-X. Winklhofer (Eds.), Neuroimaging techniques in clinical practice: Physical concepts and clinical applications (pp. 215 – 228 ). Springer International Publishing . 10.1007/978-3-030-48419-4_15

